# Bifurcatriol, a New Antiprotozoal Acyclic Diterpene from the Brown Alga *Bifurcaria* *bifurcata*

**DOI:** 10.3390/md15080245

**Published:** 2017-08-02

**Authors:** Vangelis Smyrniotopoulos, Christian Merten, Marcel Kaiser, Deniz Tasdemir

**Affiliations:** 1 School of Chemistry, National University of Ireland Galway, University Road, Galway, Ireland; vsmy@hotmail.com; 2 Lehrstuhl für Organische Chemie 2, Ruhr-Universität Bochum, Universitätsstraße 150, 44801 Bochum, Germany; christian.merten@ruhr-uni-bochum.de; 3 Swiss Tropical and Public Health Institute, 4051 Basel, Switzerland; marcel.kaiser@unibas.ch; 4 University of Basel, 4003 Basel, Switzerland; 5 GEOMAR Centre for Marine Biotechnology (GEOMAR-Biotech), Research Unit Marine Natural Product Chemistry, Research Division Marine Ecology, GEOMAR Helmholtz Centre for Ocean Research Kiel, Am Kiel-Kanal 44, 24106 Kiel, Germany

**Keywords:** *Bifurcariabifurcata*, diterpene, marine alga, VCD, absolute configuration, antiprotozoal

## Abstract

Linear diterpenes that are commonly found in brown algae are of high chemotaxonomic and ecological importance. This study reports bifurcatriol (**1**), a new linear diterpene featuring two stereogenic centers isolated from the Irish brown alga *Bifurcaria* *bifurcata*. The gross structure of this new natural product was elucidated based on its spectroscopic data (IR, 1D and 2D-NMR, HRMS). Its absolute configuration was identified by experimental and computational vibrational circular dichroism (VCD) spectroscopy, combined with the calculation of ^13^C-NMR chemical shielding constants. Bifurcatriol (**1**) was tested for in vitro antiprotozoal activity towards a small panel of parasites (*Plasmodium* *falciparum*, *Trypanosoma* *brucei* *rhodesiense*, *T.* *cruzi*, and *Leishmania* *donovani*) and cytotoxicity against mammalian primary cells. The highest activity was exerted against the malaria parasite *P.* *falciparum* (IC_50_ value 0.65 μg/mL) with low cytotoxicity (IC_50_ value 56.6 μg/mL). To our knowledge, this is the first successful application of VCD and DP4 probability analysis of the calculated ^13^C-NMR chemical shifts for the simultaneous assignment of the absolute configuration of multiple stereogenic centers in a long-chain acyclic natural product.

## 1. Introduction

Acyclic terpenes comprise a very small yet fundamental fraction of terpenes, the largest class of natural products with the highest structural and stereochemical diversity [1]. Terpene biosynthesis involves them as intermediates originating from enzymatic elongation and modification of few universal linear and non-chiral isoprenoid precursors [2,3]. The immense chemodiversity of terpenes finally emerges through a more evolved enzymatic machinery of additional cyclization and rearrangements as compared to the pathways of their acyclic primordial congeners [2,4]. Linear terpenes also encompass various modifications of the simple parent acyclic carbon skeletons. They display numerous ecological roles (e.g., defensive or communicational) and are represented in all terpene classes, both in terrestrial and marine macro- and micro-organisms. The properties and activities associated with these roles underline their great significance in industry, agriculture, and medicine [5,6,7].

Marine algal metabolites account for 13% of marine natural products, with terpenoids prevailing at a percentage over 50% [8,9]. Geranylgeraniol derivatives comprise some of the earliest compounds isolated from the order Fucales that comprise about a third of brown algal metabolites [10]. Recent literature surveys on the family Sargassaceae indicate *Bifurcaria bifurcata* (Velley) R. Ross to be one of the most prolific sources of bioactive secondary metabolites. This species accounts for 8% of the total metabolites of the family, with acyclic diterpenes being the main constituents of the organic extracts [11,12]. Many of the geranylgeraniol-derived linear diterpenes are of high chemotaxonomic and ecological importance with demonstrated antifouling, antimicrobial, antimitotic, and cytotoxic activities [11]. Unfortunately, unambiguous assignment of the absolute configuration of all stereogenic centers in many linear diterpenes has not yet been accomplished due to their molecular features [11].

It is commonly accepted that chirality, an amplifier of molecular diversity, has triggered evolution in nature by rendering most physiological interactions regio- and stereospecific [13,14]. Bioactive secondary metabolites are principally chiral compounds, necessitating the unambiguous determination of their absolute configuration (AC) and conformations, in order to gain insights into their mode of action or biosynthetic origin [14,15]. The most frequently used methods for designation of the AC in chiral molecules to date are NMR anisotropy and X-ray crystallography. However, their application in natural products is not always feasible. The alternation of NMR signals requires the use of a chiral anisotropy reagent while the formation of a single crystal is a prerequisite for X-ray crystallography [13]. The use of nondestructive chiroptical methods, such as electronic circular dichroism (ECD) and vibrational circular dichroism (VCD), as alternatives has recently gained popularity. In particular, the combination of stereochemical sensitivity with the rich structural content of vibrational spectra by VCD and Raman Optical Activity (ROA) has brought forward very reliable approaches for the AC determination of natural products [16,17]. In addition, the conformational sensitivity of vibrational spectroscopy allowed some insights into very interesting conformational variations in natural products [18] as well as peptides and other chiral molecules [19,20,21].

**Figure 1:**
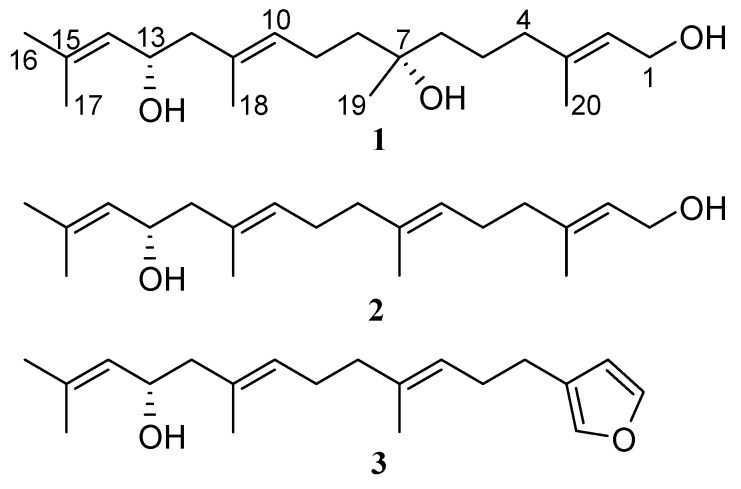
Structure of compounds (**1**–**3**) obtained from *B. bifurcata*.

In continuation of our investigation of chemical constituents of seaweeds, we isolated a new linear diterpene bifurcatriol (**1**, Figure 1) from the Irish brown alga *Bifurcaria bifurcata*. In this paper, we describe its isolation, structure elucidation, including AC assignment, and in vitro antiprotozoal activity. Encouraged by the recent successful determination of the AC of two linear diterpenes, elegandiol (**2**) and bifurcane (**3**) with a single chiral carbon (C-13) from the same alga by VCD spectroscopy [22], we explored the applicability of this method to linear diterpenes bearing multiple stereogenic centers. The VCD-based AC assignment is further supported by ^13^C-NMR chemical shift calculations [23].

## 2. Results

### 2.1. Isolation and Structure Elucidation

Algal fronds of *B. bifurcata* were collected from the shore of Kilkee, County Clare in western Ireland. The freeze-dried alga was successively extracted with CH_2_Cl_2_ and MeOH. A modified solvent partition of the combined organic residues afforded the *n*-hexane, CHCl_3_, and aqueous subextracts. The CHCl_3_ subextract was subjected to repeated automated Flash and HPLC chromatography to yield pure bifurcatriol (**1**).

Bifurcatriol (**1**) was obtained as an optically active oil. Based on its HRESIMS, its molecular formula was identified as C_20_H_36_O_3_, indicating three degrees of unsaturation. Detailed analyses of ^1^H, ^13^C-NMR, and *g*HSQC data (Table 1 and Figures S2, S3 and S5) revealed the presence of three olefinic methines (*δ*_H_/*δ*_C_ 5.39/123.6, 5.25/128.6, 5.13/127.5) and three quaternary sp^2^ carbons (*δ*_C_ 131.6, 134.8, 139.5), along with four olefinic methyl groups (*δ*_H_/*δ*_C_ 1.69/25.7, 1.66/18.2, 1.65/16.1, 1.65/16.2) and one aliphatic methyl (*δ*_H_/*δ*_C_ 1.14/26.7). The IR spectrum displayed an intense absorption band at *v*_max_ 3354 cm^−1^, characteristic for the presence of hydroxyl functionality (O–H stretch). This data was in accordance with the NMR signals attributable to an oxygenated methylene (*δ*_H_/*δ*_C_ 4.13/59.3), and an oxymethine (*δ*_H_/*δ*_C_ 4.41/65.9) group, in concurrence with a quaternary oxygen bearing carbon (*δ*_C_ 72.7). The ^1^H and ^13^C-NMR spectra also exhibited the resolved resonances of six methylenes (*δ*_H_/*δ*_C_ 2.00/39.9, 1.45/22.0, 1.40/41.7, 1.49/41.3, 2.07/22.8, 2.11/48.1), thus indicating an acyclic diterpene skeleton.

The assignment of four isolated spin systems in **1** (Figure S1) was provided by chemical shift and coupling constant data, combined with the ^3^*J*_(H-H)_ and long-range *g*COSY correlations (Table 1, Figure S4) observed between H-1/H-2, H-1/H_3_-20 and H-2/H_3_-20 for partial structure C-1/C-2/C-3/C-20, between H_2_-4/H_2_-5 for segment C-4/C-5; between H_2_-8/H_2_-9, H_2_-9/H-10, and H-10/H_3_-18 for spin system C-8/C-9/C-10/C-11/C-18; and between H_2_-12/H-13, H-13/H-14, H-14/H_3_-16, and H-14/H_3_-17 for fragment C-12/C-13/C-14/C-15/C-16(C-17). The elucidation of the planar structure shown for **1**, confidently established by connecting these segments, was based on the heteronuclear couplings observed in the *g*HMBC spectrum (Figures S1 and S6) that were also used to verify the structure of the fragments themselves. The correlations of methylene protons H_2_-4 with olefinic carbons C-2, C-3, and vinyl methyl C-20—along with those of C-4 with H_3_-20 and the olefinic proton H-2—confirmed the connection of carbons C-3 and C-4. Heteronuclear long-range couplings between C-4 and C-5 with H_2_-6, and also of methylene carbon C-6 with H_2_-4 and H_2_-5 protons, secured the connectivity of C-5 with C-6. HMBC correlations were observed from H_3_-19 to C-6 (*δ*_C_ 41.7), the oxygenated carbon C-7 (*δ*_C_ 72.7) and C-8 (*δ*_C_ 41.3), as well as between C-19/H_2_-6 and C-19/H_2_-8. Additional cross-peaks between C-7 and three methylene groups (H_2_-5, H_2_-6, and H_2_-8), established the linkage of C-8 and C-6 through the carbinol carbon atom, C-7. Finally, diagnostic *g*HMBC couplings between H_2_-12/C-10, H_2_-12/C-11, and H_2_-12/C-18; between H-10/C-12 and C-12/H_3_-18; and finally between C-11 and H-13 established the connection of methylene C-12 and quaternary olefinic C-11. This allowed the completion of the gross structure of **1** as a new acyclic diterpene bearing three OH groups at C-1, C-7, and C-13 (Figure S1), the latter two carbons being chiral.

Extended NOESY experiments in conjunction with ^1^H and ^13^C-NMR chemical shifts and coupling constants interpretation failed to elucidate the overall relative stereochemistry of **1**. However, the assignment of the double bond configurations at Δ^2^ and Δ^10^ with the *E*-geometry was secured by prominent spatial NOE correlations observed between H-2/H_2_-4 and H_2_-1/H_3_-20 (for Δ^2^), as well as H-10/H_2_-12 and H-10/H_2_-8 and H_3_-18/H-13 (for Δ^10^). The designation of H_3_-16 as pro-*E* and H_3_-17 as pro-*Z* was based on the correlations between H_3_-16 and olefinic H-14, and between H_3_-17 and H-13 (Figure S1). The adopted geometries were also supported by the relative upfield chemical shifts of the corresponding vinylic methyl groups (C-20: *δ*_C_ 16.2, C-18: *δ*_C_ 16.1, C-17: *δ*_C_ 18.2).

### 2.2. Absolute Configuration Assignment by VCD and ^13^C-NMR Spectroscopy

**Figure 2:**
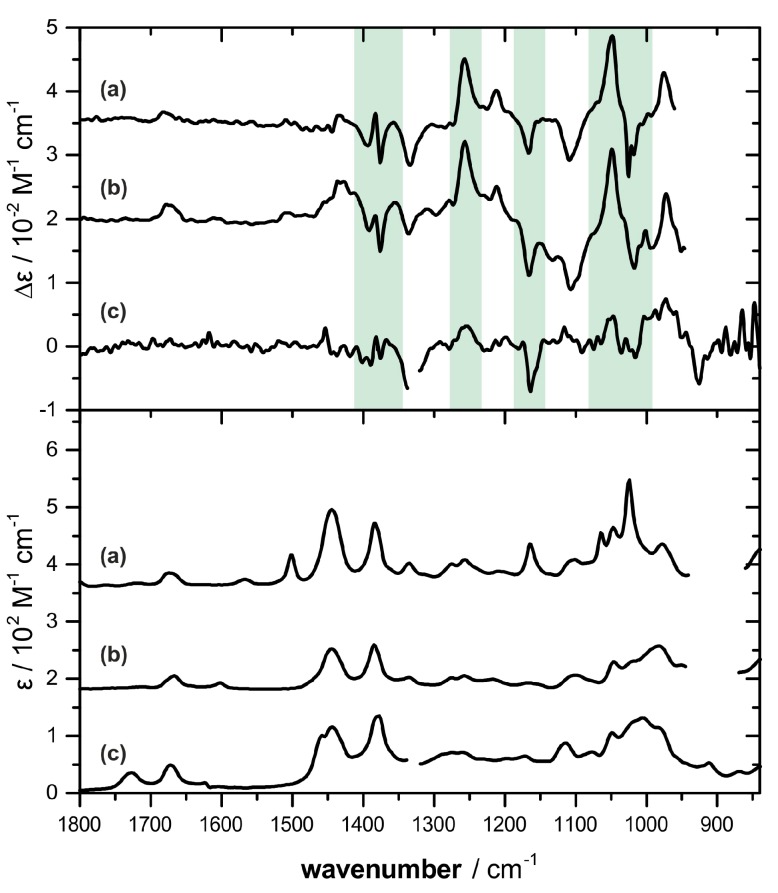
Comparison of the VCD (top) and IR (bottom) spectra of bifurcatriol (**1**) (traces c, measured in benzene-*d*_6_) against elegandiol (**2**) (traces b, CDCl_3_) and bifurcane (**3**) (traces a, CDCl_3_) reported previously [22]. The green shaded VCD signatures appear to be characteristic for the C-13 stereocenter. Spectral regions around 1340 cm^−1^ (traces c) and between 950–870 cm^−1^ (traces a and b) are left out due to solvent absorbance.

Based on our previous experience, concentrations of 0.5 M are necessary in order to achieve good signal to noise ratio for a VCD-based AC assignment. Bifurcatriol (**1**) was in that sense a challenging sample, as only a few milligrams were available. Nevertheless, we recorded the IR and VCD spectra of **1** for a solution of the available 4.7 mg in 60 μL of benzene-*d*_6_, which resulted in a concentration of 0.24 M. The obtained spectra are compared to the previously recorded spectra of elegandiol (**2**) and bifurcane (**3**) [22] shown in Figure 2. Although more noisy, some characteristic similarities with **2** and **3** can be identified in the spectra of **1**, highlighted green in Figure 2. In particular, the strong negative VCD band at 1153 cm^−1^ and the onset of the positive bands at 1255 and 1050 cm^−1^ suggest the possibility of the same (13*S*)-configuration in **1** as for the previously characterized linear diterpenes **2** and **3**.

Assuming a (13*S*)-configuration, we then calculated IR and VCD spectra for (7*S*,13*S*)-**1** and (7*R*,13*S*)-**1**. The generation of the structures of both stereoisomers were followed by a conformational search on a force field level (MMFF using Spartan 14 [24], Monte-Carlo approach). The 100 lowest energy structures of both sets of optimized geometries were subjected to further geometry optimization at density functional theory (DFT) level at the B3LYP/6-31G(2d,p)/IEFPCM (benzene) level of theory. Although the MMFF conformational searches for both stereoisomers were repeated for several initial geometries, less than 10 conformers were found to account for ~95% of the conformation population after the DFT optimizations. Therefore, the 20 lowest energy structures obtained from this DFT-based pre-optimization were submitted to another optimization cycle at the B3LYP/6-311++G(2d,p)/IEFPCM (benzene) level of theory. The final computed IR and VCD spectra were obtained by Boltzmann-averaging of the single-conformer spectra according to the populations calculated for ΔG_298K_.

**Figure 3:**
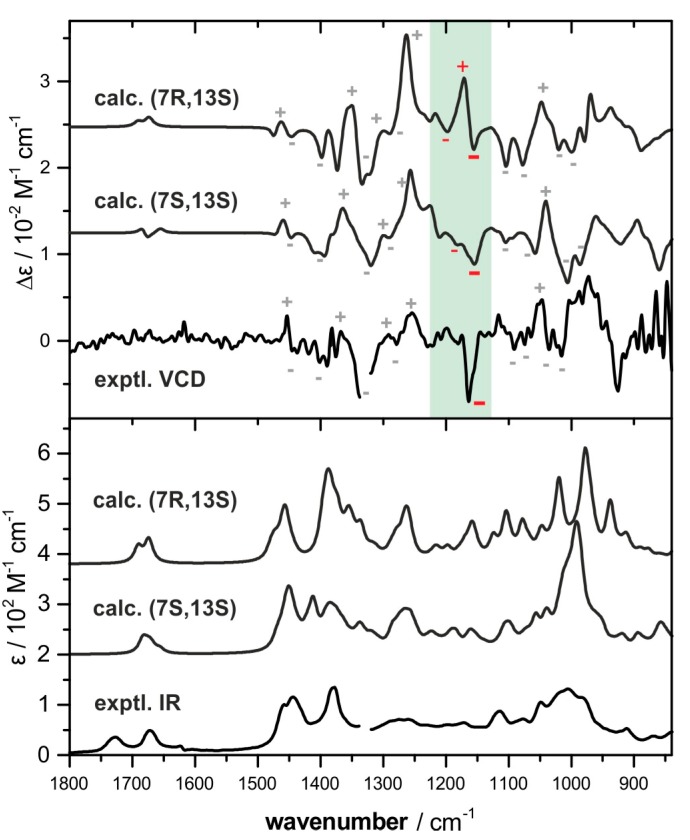
Comparison of the VCD (top) and IR (bottom) spectra of bifurcatriol **1** (measured in benzene-*d*_6_) with those calculated for (7*S*,13*S*)-**1** and (7*R*,13*S*)-**1**. The green shaded VCD signatures appear to be characteristic for the stereocenter at C-7. Spectral regions around 1340 cm^−1^ and below 900 cm^−1^ are left out due to solvent absorbance and high noise.

In comparison with the experimental spectra, both sets of spectra agree moderately owing to the high noise level of the experimental VCD spectrum. As indicated in Figure 3, the calculated spectral features in the ranges 1400–1230 cm^−1^ and 1050–1000 cm^−1^ show very similar pattern, so that both confirm the (13*S*)-configuration of **1**. In turn, the computed VCD spectra of the two possible stereoisomers feature only very few bands, which are suited to differentiate between them. Mainly, this is the case in the spectral range from 1230 to 1130 cm^−1^, in which the (7*S*,13*S*)-isomer features generally weaker and mostly negative bands while the (7*R*,13*S*)-isomer features a strong positive component at ~1170 cm^−1^. When examining the single-conformer spectra of the main conformers (Figure S10), this difference in the band pattern can be assumed to be characteristic for the C7-stereocenter, as it is found to be essentially constant in all spectra. Based on the lack of this strong positive feature, and the qualitatively better agreement of the other VCD spectral signatures as well as of the IR spectral pattern, the absolute configuration of **1** was tentatively assigned to be (7*S*,13*S*).

In order to further support this assignment, we took advantage of a probability measure recently developed by Goodman and co-workers [25]. This so-called DP4 probability is capable of distinguishing between two or more diastereomers (but not enantiomers) based on a comparison of calculated NMR chemical shifts with experimental data (Figure S11). For this comparison, it is necessary to calculate the NMR shielding constants for every conformer, and subsequently determine the chemical shifts for each atom by Boltzmann averaging over the single-conformer values (Table S1). The GIAO NMR shift calculation is integral part of a VCD intensity calculation, and thus these values are usually available after the VCD spectral analysis. Combining an IR/VCD spectroscopic analysis can thus easily be supplemented by the DP4 probability [26]. In the present case, however, the NMR and vibrational spectra were recorded in different solvents, so that the geometries of **1** used to simulate the IR and VCD spectra were subjected to another cycle of optimization and GIAO NMR shift calculation employing the IEFPCM of benzene. Afterwards, the predicted ^13^C-NMR shifts shown in Table S2 were obtained by referencing the obtained shielding constants to the shielding constant of TMS, which has been calculated at the same level of theory. These numbers were then submitted to the web applet for the calculation of DP4 probabilities provided by Goodman et al. [25] which gave a 100% confidence for **1** having a (7*S*,13*S*)-configuration. This high confidence of the algorithm certainly has to be interpreted with caution, as there is not yet much data on its performance on highly flexible molecules. However, in light of the fact that it agrees with the conclusion drawn from the VCD analysis, we see it as confirmation. Hence the structure of bifurcatriol was identified as (2*E*,7*S*,10*E*,13*S*)-3,7,11,15-tetramethylhexadeca-2,10,14-triene-1,7,13-triol.

### 2.3. Bioactivity

Bifurcatriol (**1**) was evaluated for its in vitro antiprotozoal activity: towards a panel composed of *Plasmodium falciparum*, *Trypanosoma brucei rhodesiense*, *T. cruzi*, and *Leishmania donovani*; and against L6 primary rat myoblast cells to assess its general cytotoxicity. As shown in Table 2, **1** showed remarkable activity against drug resistant K1 strain of the malaria parasite, *P. falciparum* with an IC_50_ value of 0.65 μg/mL. The general toxicity against L6 cells was 56.6 μg/mL, indicating that **1** has a high selectivity index of 87. The activity against other protozoan species was only moderate, with IC_50_ values of 11.8 μg/mL (*T. brucei rhodesiense*), 47.8 μg/mL (*T. cruzi*), and 18.8 μg/mL (*L. donovani*). This indicates that **1** has specific antiplasmodial activity with good selectivity.

## 3. Discussion

Determination of the AC of natural products is crucial for their bioactivity/toxicity, feasibility for organic synthesis, and identification of biogenetic origin, yet it remains mostly very demanding or problematic [15]. The major challenge in the structural elucidation of the linear diterpenes isolated from *B. bifurcata* and other members of the Sargassaceae family rests in the determination of the AC of their oxidized stereocenters. Due to the inherent incapacity of these compounds to shape crystals suitable for X-ray crystallographic analyses, the most frequently used method for AC determination is Horeau’s and Mosher’s derivatization approaches. However, the proximity of additional hydroxyls, difficulties in the assignment of diagnostic derivative protons, or steric hindrances in asymmetric quaternary carbon environments—as in the case of **1**—renders the NMR anisotropy method insufficient [10,11]. The absolute configuration of **1** has been assigned based on the results of VCD spectroscopy and DP4 probability analyses of the calculated ^13^C-NMR chemical shifts. The combination of two techniques for the assignment was necessary, as the available amounts of **1** did not allow us to measure the VCD spectrum at an optimum concentration. As a result, the VCD spectrum was rather noisy and did not allow for an unambiguous assignment. The DP4 analysis, however, allowed us to clearly distinguish the diastereomers based on the predicted ^13^C-NMR shifts, and thus to confirm the structure of the target compound. The present study thus showcases the power of joining methods for the assignment of AC [27]. To our knowledge, **1** is the first acyclic natural product with two stereogenic centers whose AC assignment was simultaneously and successfully determined via a combined VCD and DP4/NMR analyses without further derivatization. Furthermore, bifurcatriol (**1**) appears to be a lead compound whose antimalarial activity can be improved by medicinal chemistry approaches.

## 4. Materials and Methods

### 4.1. General Experimental Procedures

Optical rotations were determined at the sodium D line (589.3 nm), at 20 °C, with a 10 cm cell, on a Unipol L1000 Schmidt + Haensch polarimeter. UV spectra were acquired in spectroscopic grade CHCl_3_ on a Varian, Cary 100 UV–Vis spectrophotometer. IR spectra were recorded on a Perkin Elmer 400 ATR FT-IR spectrometer. NMR spectra were acquired using a Varian 500 MHz spectrometer. Chemical shifts are given on the *δ* (ppm) scale, referenced to the residual solvent signal (CDCl_3_: *δ*_H_ 7.24, *δ*_C_ 77.0), and coupling constant values (*J*) are reported in Hz. High resolution mass spectrometric data were measured on an Agilent 1290 Infinity UPLC system, operating on the elution gradient: 50% B for 8 min, increasing to 100% B in 3 min, maintaining 100% B for 5 min (solvent A: H_2_O + 0.1% formic acid, solvent B: MeCN + 0.1% formic acid), on a ZORBAX Eclipse Plus RRHD C18 (50 × 2.1 mm, 1.8 μm) column, flow 0.5 mL/min, UV detection 200–600 nm, combined with an Agilent 6540 QTOF system, with electrospray ionization (ESI) in the positive ion mode. Thin layer chromatography was performed on silica gel 60F_254_ supported on aluminum sheets (Merck, Tullagreen, Ireland). Substances were detected by UV at 254 and 366 nm or after spraying with 6% vanillin and 15% H_2_SO_4_ in MeOH reagents and charring. All solvents were of HPLC or LC-MS grade and were purchased from Sigma-Aldrich (Arklow, Ireland).

### 4.2. Algal Material

*Bifurcaria bifurcata* was collected by hand from the intertidal rock pool at Kilkee, County Clare of Ireland, in May of 2009 and kept frozen until the work-up. A voucher specimen (BDV0015) is kept at the Herbarium of the Biodiscovery Laboratory in the Irish Marine Institute.

### 4.3. Extraction and Isolation

The freeze-dried algal material (132.4 g dry weight) was exhaustively extracted at room temperature consecutively with CH_2_Cl_2_ and MeOH. The combined extracts were concentrated to give a dark green residue (12.0 g) that was subjected to a modified Kupchan partition scheme. Briefly, the crude extract was partitioned between 10% aqueous MeOH and *n*-hexane. The water concentration of the aq. MeOH phase was increased to 35%, before partitioning against CHCl_3_. Evaporation of the solvent under reduced pressure afforded the CHCl_3_ subextract (7.6 g), which was subjected to automated Flash chromatography fractionation on an Agilent 971FP system loaded with a pre-packed Varian SuperFlash Si50 SF25-80g silica column (277 × 28.2 mm, 50 μm), operating the gradient: 0% B for 5 min, increasing to 5% B in 15 min, maintaining 5% B for 10 min, increasing to 10% B in 10 min, maintaining 10% B for 10 min, increasing to 40% B in 40 min, increasing to 100% B in 10 min, maintaining 100% B for 10 min (solvent A: *n*-hexane, solvent B: EtOAc), at a flow of 25 mL/min, collecting 25 mL fractions. Combined fractions 63 and 64 that eluted with 80% EtOAc in *n*-hexane (19.5 mg) were subjected to RP-HPLC. The separation was conducted using an Agilent 1260 HPLC system equipped with a diode array and an ELSD detector (split flow) on a Kromasil 100 C18 (250 × 8 mm, 5 μm) HPLC reversed-phase column. The elution gradient was: 55% B for 13 min, increasing to 100% B in 5 min, maintaining 100% B for 20 min (solvent A: H_2_O, solvent B: MeCN), at a flow of 1.5 mL/min, and afforded **1** in pure state (7.7 mg, 0.007%, *t*_R_ 16.3 min).

*Bifurcatriol* (**1**): colorless oil; ${\left[\alpha \right]}_{D}^{25}$ −3.0 (*c* 0.6, CHCl_3_); UV (CHCl_3_) *λ*_max_ (log ε) 245 (2.31) nm; IR (film) *ν*_max_ 3354, 2968, 2930, 1670 cm^−1^; ^1^H-NMR (CDCl_3_, 500 MHz) and ^13^C-NMR (CDCl_3_, 125 MHz) see Table 1; HRMS (ESI-TOF) *m*/*z* 347.2559 [M + Na]^+^ (calcd for C_20_H_36_O_3_Na, 347.2557).

### 4.4. IR and VCD Spectroscopy

The IR and VCD spectra were recorded on a Bruker Vertex 70 V spectrometer equipped with a PMA 50 module for polarization modulated measurements. Samples were held in a sealed BaF_2_ IR cell with 100 μm path length. Both IR and VCD spectra were recorded at 4 cm^−1^ spectral resolution by accumulating ~20,000 scans (4 h accumulation time for VCD). Baseline correction of the spectra was done by subtraction of the solvent spectra measured under identical conditions.

### 4.5. Computational Analysis

Force field (MMFF94) based conformational analysis of **1** was carried out using the implemented algorithms of Spartan 14 [24]. DFT-based geometry optimizations and spectra calculations were carried out using Gaussian 09 D.01 [28] employing the B3LYP functional and the 6-31G(2d,p) and 6-311++G(2d,p) basis set. All calculations were carried out considering implicit solvation with a polarizable continuum model for benzene and chloroform. Lorentzian band shapes with 8 cm^−1^ half-width at half height were assigned to the calculated dipole and rotational strength, and the frequencies were scaled by a factor of 0.975 for better visual comparison.

### 4.6. Antiprotozoal Activity

The in vitro antiparasitic activities of **1** (Table 2) against *P. falciparum* (K1 strain), *T. cruzi*, *T. brucei rhodesiense*, *L. donovani*, and the cytotoxicity against rat skeletal myoblast L6 cells were determined as described previously [29].

## Figures and Tables

**Table 1:** NMR spectroscopic data (500 MHz, CDCl_3_) of bifurcatriol (**1**)

Position	*δ*_C_, Type	*δ*_H_, Mult (*J* in Hz)	COSY	NOESY	HMBC (^1^H → ^13^C)
1	59.3, CH_2_	4.13, d (6.8)	2, 20	20	2, 3
2	123.6, CH	5.39, t (6.8)	1, 20	4	1, 4, 20
3	139.5, C				
4	39.9, CH_2_	2.00, t (7.0)	5	2	2, 3, 5, 6, 20
5	22.0, CH_2_	1.45, m	4	19	3, 4, 6, 7
6	41.7, CH_2_	1.40, m		19	4, 5, 7, 8, 19
7	72.7, C				
8	41.3, CH_2_	1.49, t (7.8)	9	10, 19	6, 7, 9, 10, 19
9	22.8, CH_2_	2.07, dt (7.0, 7.8)	8, 10	19	7, 8, 10, 11
10	128.6, CH	5.25, t (7.0)	9, 18	8, 12	8, 9, 12, 13, 18
11	131.6, C				
12	48.1, CH_2_	2.11, d (6.9)	13	10, 14	10, 11, 13, 14, 18
13	65.9, CH	4.41, dt (8.2, 6.9)	12, 14	17, 18	11, 12, 14, 15
14	127.5, CH	5.13, d (8.2)	13, 16, 17	12, 16	12, 13, 16, 17
15	134.8, C				
16	25.7, CH_3_	1.69, br.s	14	14	14, 15, 17,
17	18.2, CH_3_	1.66, br.s	14	13	14, 15, 16,
18	16.1, CH_3_	1.65, br.s	10	13	10, 11, 12
19	26.7, CH_3_	1.14, s		5, 6, 8, 9	6, 7, 8
20	16.2, CH_3_	1.65, br.s	2	1	2, 3, 4

**Table 2:** In vitro antiprotozoal activity of bifurcatriol (**1**). IC_50_ values are in μg/mL. Reference compounds ^a^ chloroquine, ^b^ melarsoprol, ^c^ benznidazole, ^d^ miltefosine, ^e^ podophyllotoxin. Cytotoxicity was evaluated against the L6 rat myoblast cell line.

Compound	*P. falciparum*	*T. b. rhodesiense*	*T. cruzi*	*L. donovani*	Cytotoxicity
1	0.65	11.8	47.8	18.8	56.6
Reference	0.05 ^a^	0.01 ^b^	0.59 ^c^	0.12 ^d^	0.004 ^e^

## Supplementary Materials

The following are available online at www.mdpi.com/1660-3397/15/8/245/s1, Figure S1: Key HMBC COSY and NOESY correlations observed in compound **1**, Figure S2: ^1^H-NMR spectrum (500 MHz, CDCl_3_) of compound **1**, Figure S3: ^13^C-NMR spectrum (125 MHz, CDCl_3_) of compound **1**, Figure S4: gCOSY spectrum (500 MHz, CDCl_3_) of compound **1**, Figure S5: gHSQC spectrum (500/125 MHz, CDCl_3_) of compound **1**, Figure S6: gHMBC spectrum (500/125 MHz, CDCl_3_) of compound **1**, Figure S7: NOESY spectrum (500 MHz, CDCl_3_) of compound **1**, Figure S8: ESI HR-MS report for compound **1**, Figure S9: FT-IR (ATR) spectrum of compound **1**, Figure S10: Comparison of the calculated VCD spectra of some key conformers of both possible stereoisomers, Figure S11: Correlation of experimental and predicted ^13^C-NMR chemical shifts of (7*S*,13*S*)-**1** and (7*R*,13*S*)-**1**, Table S1: Comparison of experimental and calculated ^13^C chemical shifts (CDCl3), Table S2: Calculated ^13^C-chemical shifts for (7*S*,13*S*)-**1** and (7*R*,13*S*)-**1** (b3lyp/6-311++G(2d,p)/IEFPCM/CHCl_3_).
