# Supplementary material for: Bifurcatriol, a New Antiprotozoal Acyclic Diterpene from the Brown Alga Bifurcaria bifurcata
Source: Mar Drugs. 2017 Aug 2;15(8):245. doi: 10.3390/md15080245 (PMC5577600; doi:10.3390/md15080245)
Supplement: Supplementary file 1 [file marinedrugs-15-00245-s001.pdf]

SUPPORTING INFORMATION

Bifurcatriol, a New Antiprotozoal Acyclic Diterpene from the Brown Alga *Bifurcaria bifurcata*

Vangelis Smyrniotopoulos [1], Christian Merten [2], Marcel Kaiser [3,4] and Deniz Tasdemir [1,5*]

[1] School of Chemistry, National University of Ireland Galway, University Road, Galway, Ireland; vsmy@hotmail.com
[2] Lehrstuhl für Organische Chemie 2, Ruhr-Universität Bochum, Universitätsstraße 150, 44801 Bochum, Germany; christian.merten@ruhr-uni-bochum.de
[3] Swiss Tropical and Public Health Institute, CH-4051, Basel, Switzerland; marcel.kaiser@unibas.ch
[4] University of Basel, CH-4003, Basel, Switzerland
[5] GEOMAR Centre for Marine Biotechnology (GEOMAR-Biotech), Research Unit Marine Natural Product Chemistry, Research Division Marine Ecology, GEOMAR Helmholtz Centre for Ocean Research Kiel, Am Kiel-Kanal 44, 24106 Kiel, Germany; dtasdemir@geomar.de
* Correspondence: dtasdemir@geomar.de; Tel.: ++49-431-6004430

Table of Contents

Figure S1. Key HMBC (solid line arrows), COSY (bold line) and NOESY (dashed line arrows) correlations observed in **1** 3

Figure S2. ^{1}H NMR spectrum (500 MHz, $CDCl_3$) of compound **1** 4

Figure S3. ^{13}C NMR spectrum (125 MHz, $CDCl_3$) of compound **1** 5

Figure S4. *g*COSY spectrum (500 MHz, $CDCl_3$) of compound **1**. 6

Figure S5. *g*HSQC spectrum (500/125 MHz, $CDCl_3$) of compound **1**. 7

Figure S6. *g*HMBC spectrum (500/125 MHz, $CDCl_3$) of compound **1**. 8

Figure S7. NOESY spectrum (500 MHz, $CDCl_3$) of compound **1** 9

Figure S8. ESI HR-MS report for compound **1** 10

Figure S9. FT-IR (ATR) spectrum of compound **1**. 12

Figure S10. Comparison of the calculated VCD spectra of some key conformers of both possible stereoisomers. The green shaped area used to distinguish the isomers is found to be almost unaffected by conformational changes 12

Figure S11. Correlation of experimental and predicted ^{13}C-NMR chemical shifts of (7*S*,13*S*)-**1** and (7*R*,13*S*)-**1** 13

Table S1. Comparison of experimental and calculated ^{13}C chemical shifts ($CDCl_3$).. 13

Table S2. Calculated ^{13}C-chemical shifts for (7*S*,13*S*)-**1** and (7*R*,13*S*)-**1** (b3lyp/6-311++G(2d,p)/IEFPCM/$CHCl_3$).. 13

Figure S1. Key HMBC (solid line arrows), COSY (bold line) and NOESY (dashed line arrows) correlations observed in **1**.

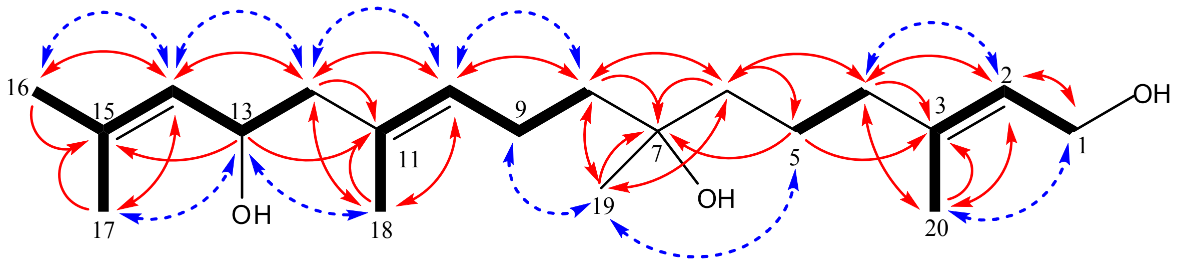

Figure S2. ^{1}H NMR spectrum (500 MHz, $CDCl_3$) of compound **1**.

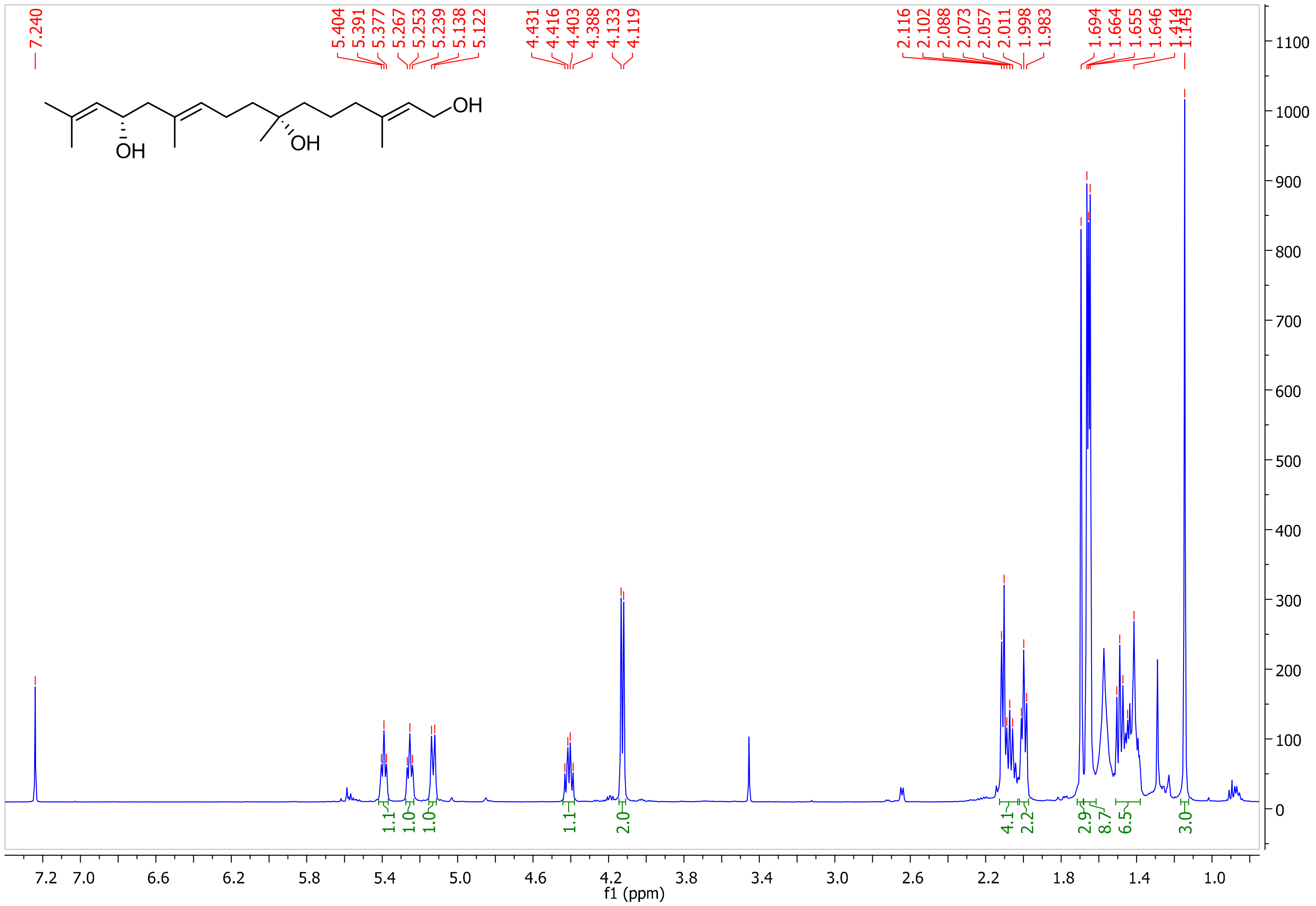

Figure S3. ^{13}C NMR spectrum (125 MHz, $CDCl_3$) of compound **1**.

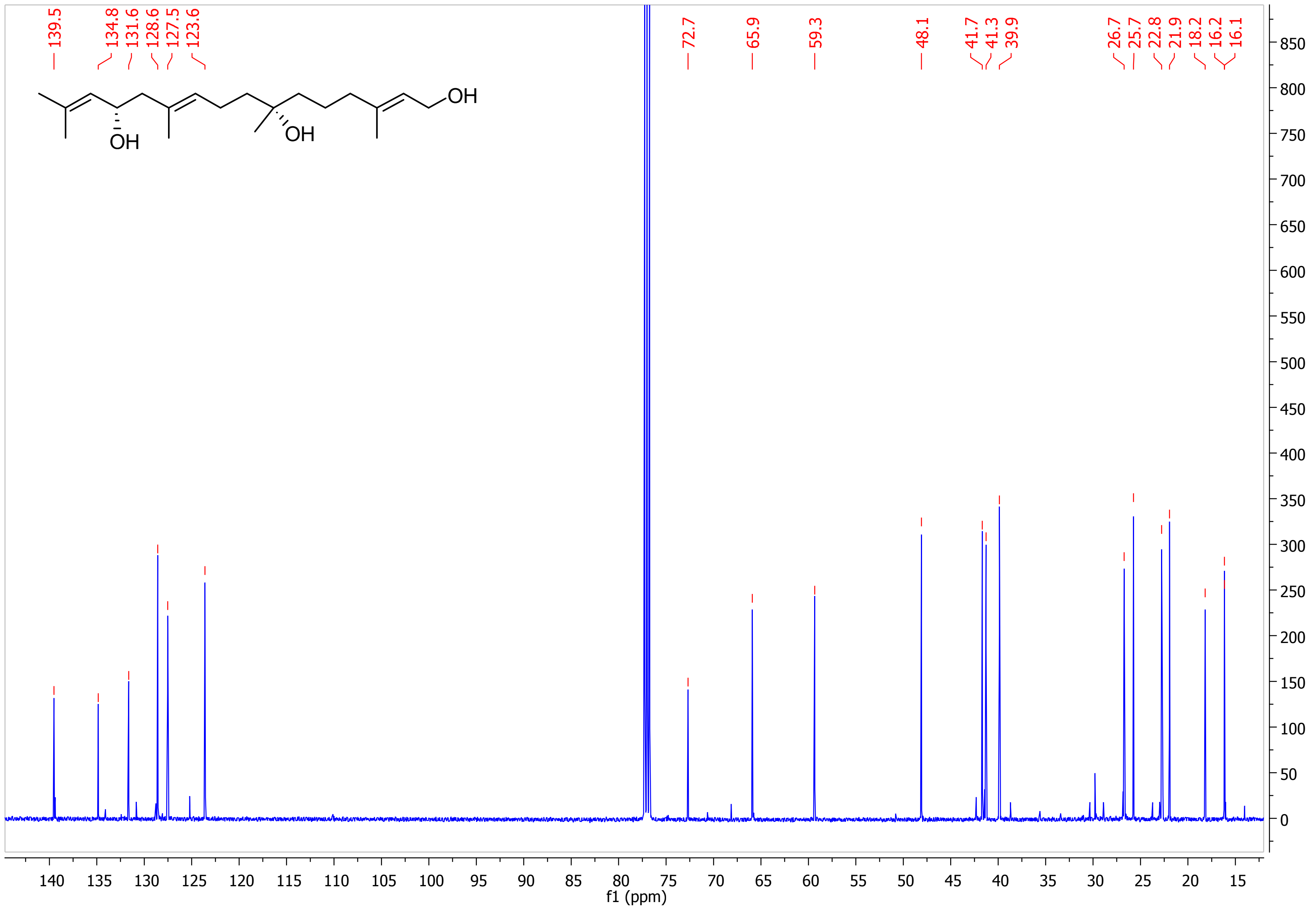

Figure S4. *g*COSY spectrum (500 MHz, $CDCl_3$) of compound **1**.

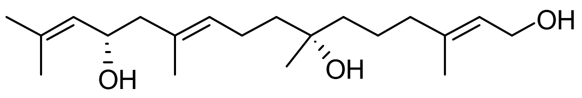

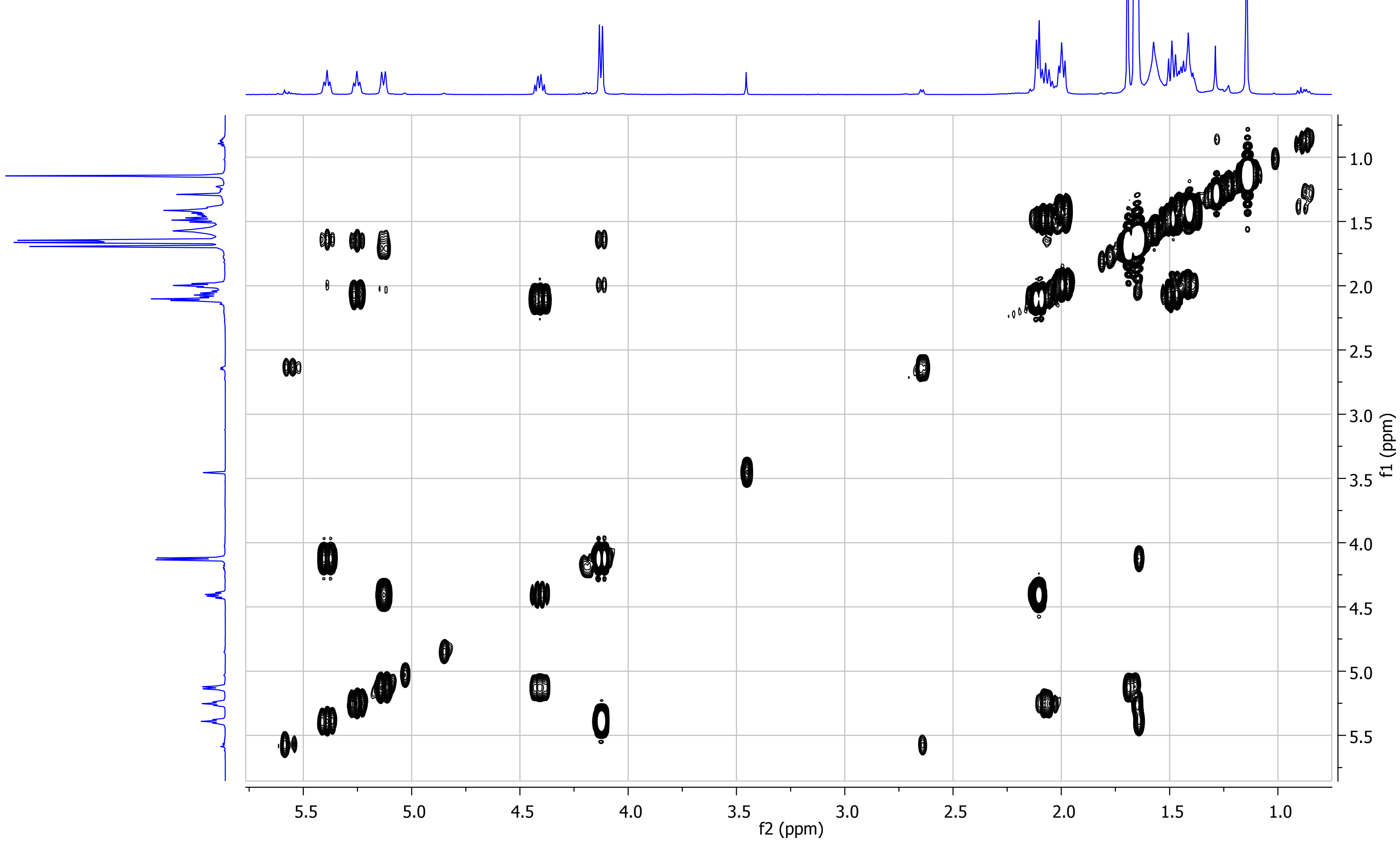

Figure S5. *g*HSQC spectrum (500/125 MHz, $CDCl_3$) of compound **1**.

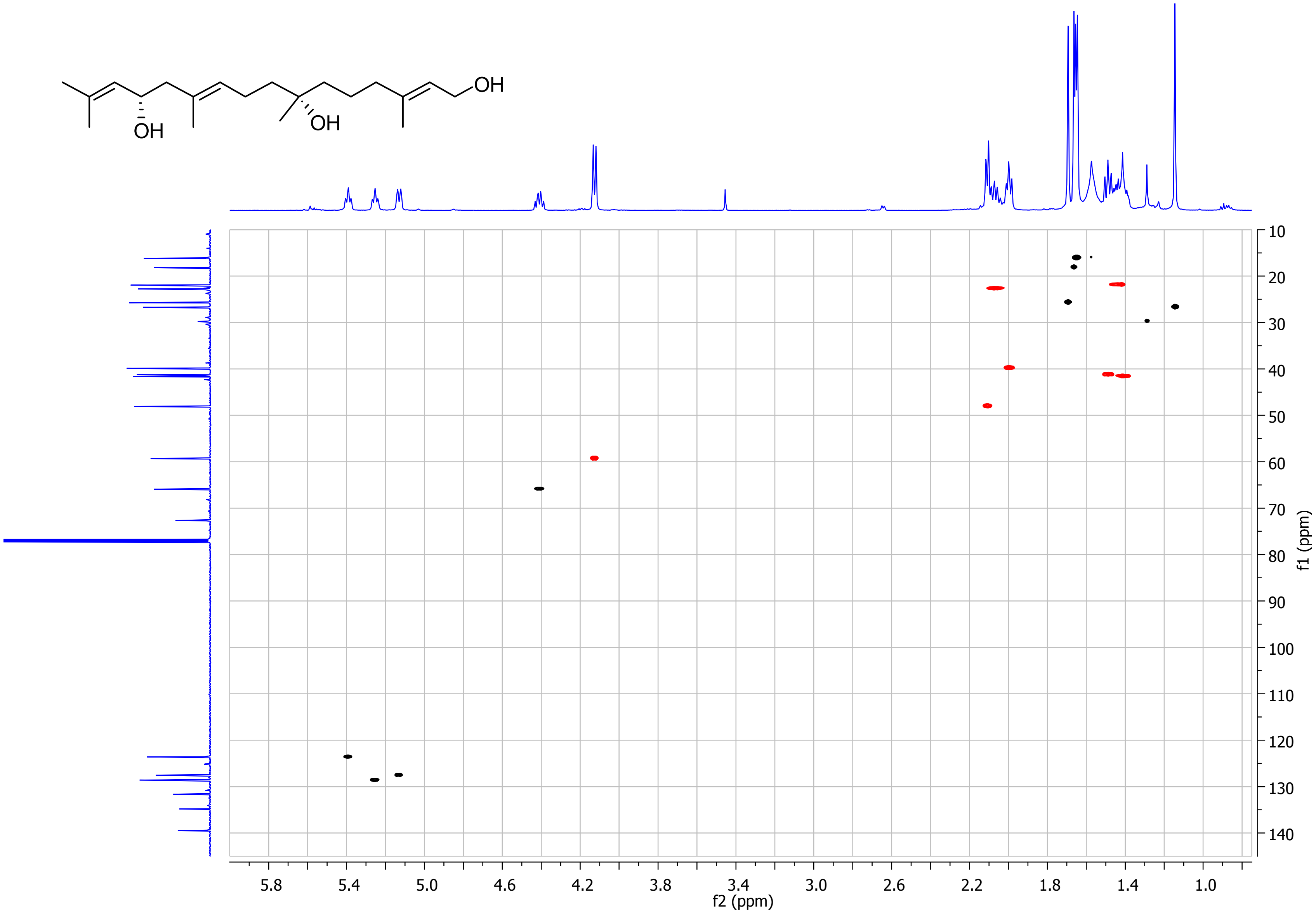

Figure S6. *g*HMBC spectrum (500/125 MHz, $CDCl_3$) of compound **1**.

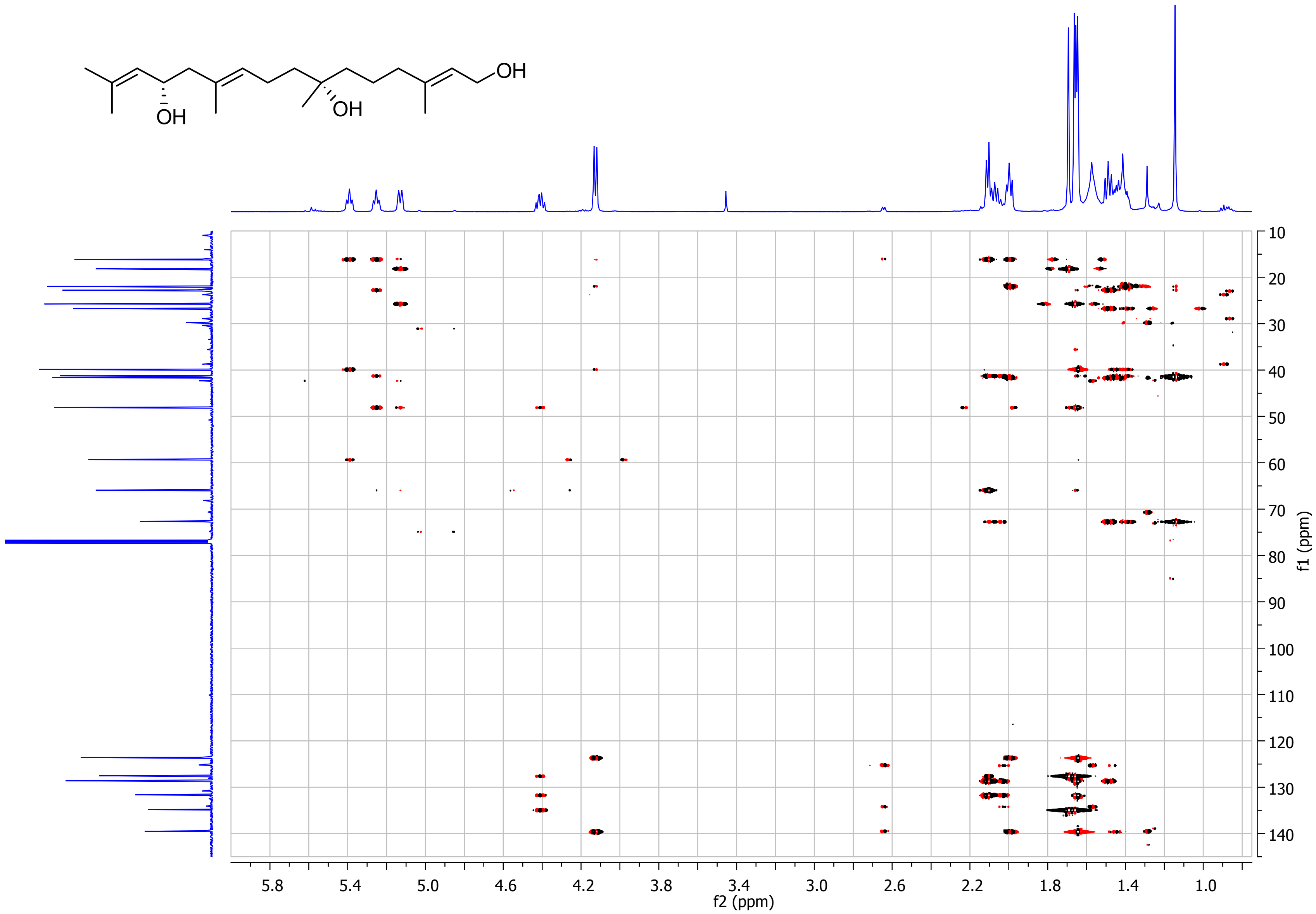

Figure S7. NOESY spectrum (500 MHz, $CDCl_3$) of compound **1**.

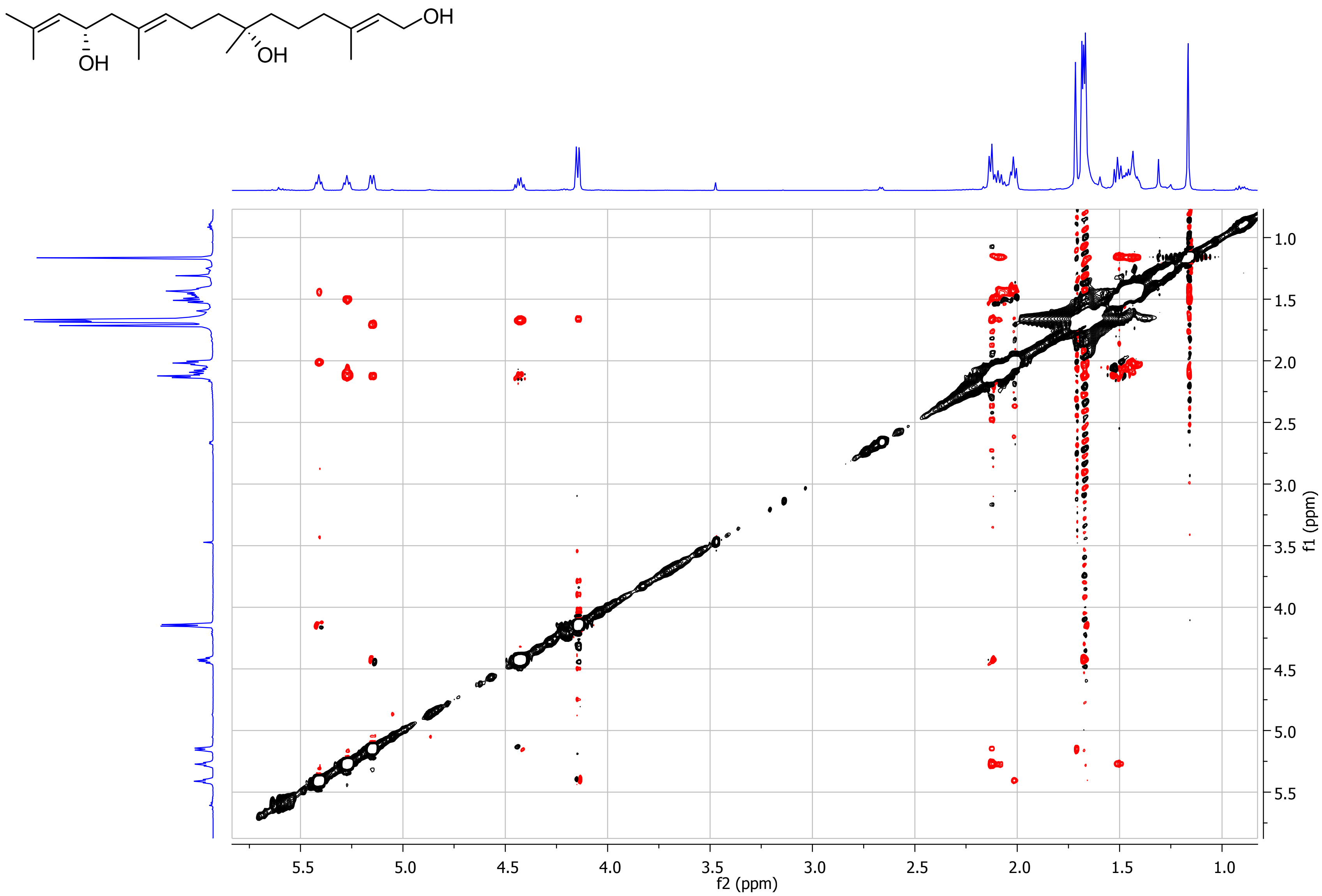

Figure S8. ESI HR-MS report for compound **1**.

**DataFile**	bifu4_15C14-2_2014-07-30_1.d	**SampleName**	15C14-2_bifu4
**SampleType**	Sample	**Position**	P1-A3
**InstrumentName**	Instrument 1	**UserName**	
**AcqMethod**	VsmyPosFragile_BDV15.m	**AcquiredTime**	30-Jul-14 21:23:54
**IRM CalibrationStatus**	Success	**DA Method**	Default.m
**Comment**			

**SampleGroup**	**Info.**
**Fragmentor**	**NozzleVoltage**

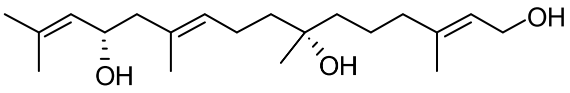

Compound Table

Compound Label	RT	Mass	Abund	Formula	TgtMass	Diff (ppm)	MFG Formula
Cpd 1:	5,59	324,2667	510741	C20H36O3	324,2664	0,83	C20H36O3

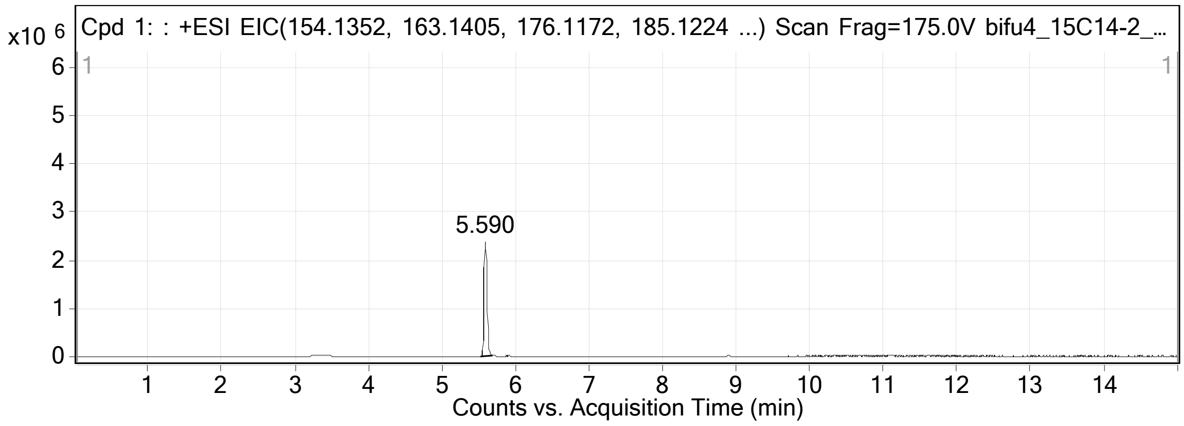

MS Spectrum

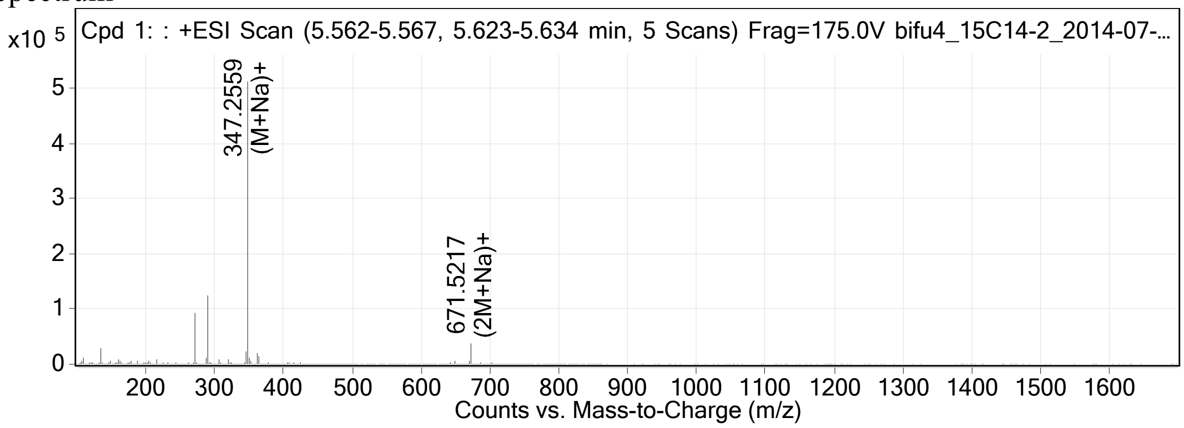

MS ZoomedSpectrum

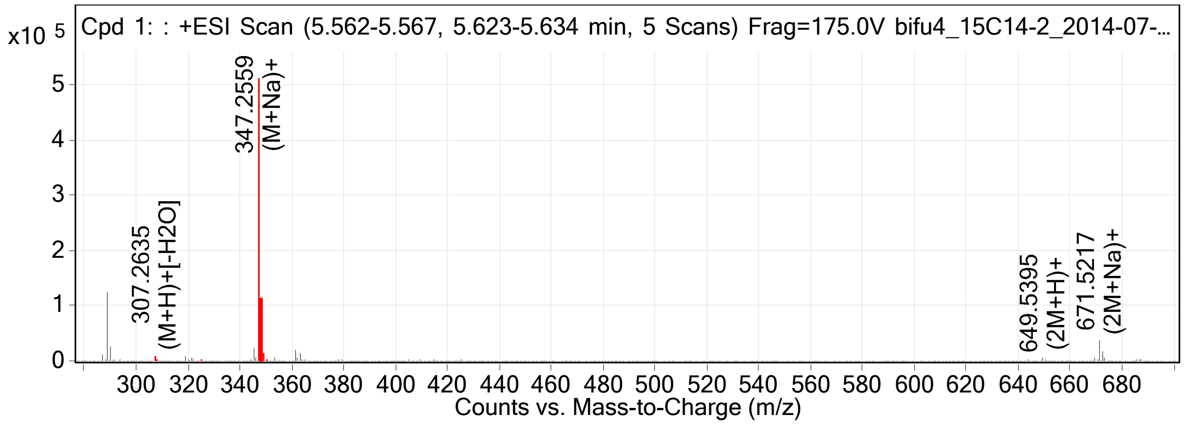

MS SpectrumPeakList

m/z	*Calc m/z*	Diff (ppm)	z	Abund	Formula	Ion
307,2635	307,2632	-1,07	1	8316,4	C20H35O2	$(M+H)^+$-H_2O
325,2739	325,2737	-0,42	1	1742,8	C20H37O3	$(M+H)^+$
347,2559	347,2557	-0,77	1	510740,9	C20H36NaO3	$(M+Na)^+$
348,2594	348,2591	-0,78	1	105320,6	C20H36NaO3	$(M+Na)^+$
349,2618	349,262	0,37	1	12928,4	C20H36NaO3	$(M+Na)^+$
631,528	631,5296	2,54	1	1036,3	C40H71O5	$(2M+H)^+$-H_2O
649,5395	649,5402	1,1	1	4832,7	C40H73O6	$(2M+H)^+$
671,5217	671,5221	0,65	1	36792,7	C40H72NaO6	$(2M+Na)^+$
672,5252	672,5255	0,41	1	15971,7	C40H72NaO6	$(2M+Na)^+$

Figure S9. FT-IR (ATR) spectrum of compound **1**.

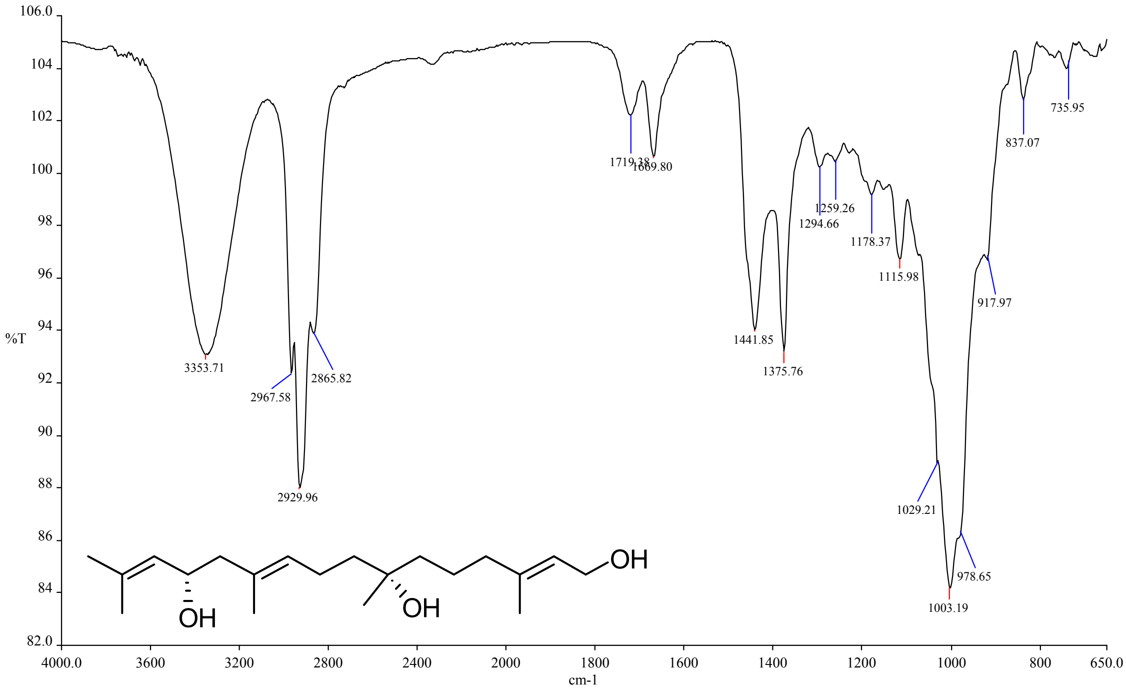

Figure S10. Comparison of the calculated VCD spectra of some key conformers of both possible stereoisomers. The green shaped area used to distinguish the isomers is found to be almost unaffected by conformational changes.

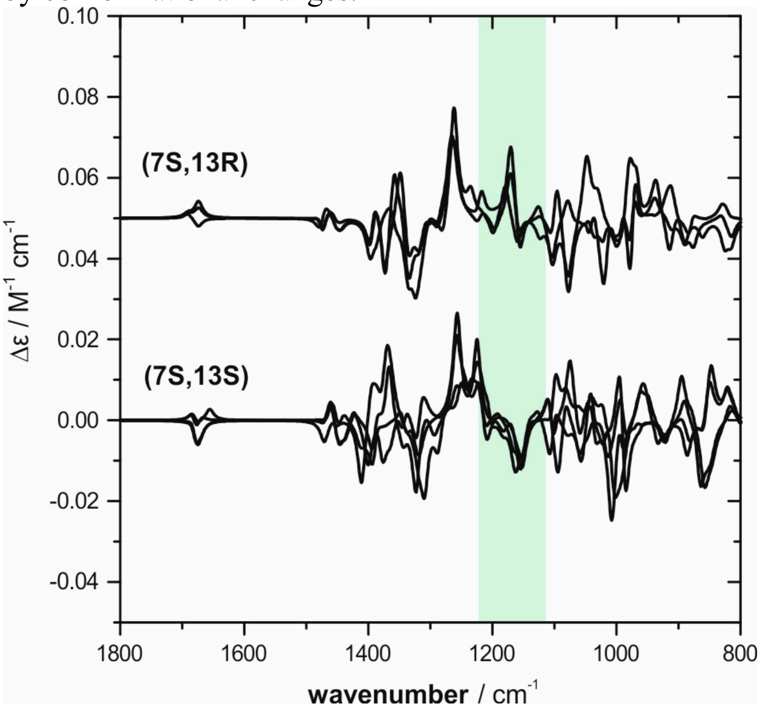

Figure S11. Correlation of experimental and predicted ^{13}C-NMR chemical shifts of (7*S*,13*S*)-**1** and (7*R*,13*S*)-**1**.

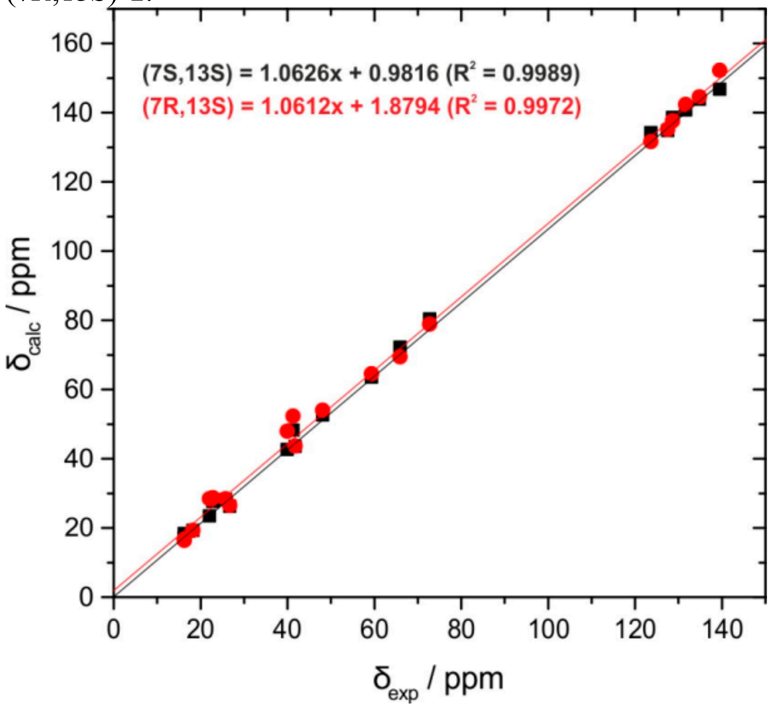

Table S1. Comparison of experimental and calculated ^{13}C chemical shifts ($CDCl_3$).

Carbon	δ_C exp	δ_C calc (7*S*,13*S*)	δ_C calc (7*R*,13*S*)
1	59.3	64.2	64.9
2	123.6	132.8	131.3
3	139.5	147.9	153.4
4	39.9	42.1	48.1
5	22.0	23.5	28.7
6	41.7	43.2	43.7
7	72.7	81.2	79.4
8	41.3	48.9	52.2
9	22.8	27.7	28.8
10	128.6	138.8	138.2
11	131.6	141.4	142.5
12	48.1	53.0	53.9
13	65.9	72.0	69.7
14	127.5	134.4	134.8
15	134.8	145.3	145.9
16	25.7	28.4	28.5
17	18.2	19.4	19.4
18	16.1	17.0	16.5
19	26.7	26.2	26.6
20	16.2	18.9	16.8
MAD[(a)]		3.0	3.2
R^{2}[(b)]		0.9987	0.9971
DP4[(c)]		100%	0%

[(a)] mean average deviation (in ppm), [(b)] square of the correlation factor, [(c)] DP4 probability [25].

Table S2. Calculated ^{13}C-chemical shifts for (7*S*,13*S*)-**1** and (7*R*,13*S*)-**1** (b3lyp/6-311++G(2d,p)/IEFPCM/$CHCl_3$).

					**Calculated ^{13}C-chemical shifts for (7S,13S)-isomer (b3lyp/6-311++G(2d,p)/IEFPCM/$CHCl_3$)**																			
	**ΔE**	**ΔG**	**pop-ΔE**	**pop-ΔG**	1	2	3	4	5	6	7	8	9	10	11	12	13	14	15	16	17	18	19	20
bifu4_isomer1_c1	0.00	0.00	34.86	19.86	62.79	136.54	151.34	47.38	23.81	47.67	80.14	48.22	28.41	141.93	137.81	53.41	72.61	134.44	145.16	28.44	19.41	16.98	23.88	16.48
bifu4_isomer1_c2	0.45	0.19	16.31	14.35	63.61	136.45	146.92	42.66	23.61	43.42	82.03	46.01	27.07	142.08	138.64	53.57	71.95	134.67	144.20	28.34	19.41	16.58	26.28	16.30
bifu4_isomer1_c3	4.05	0.30	0.04	11.92	65.22	127.37	150.03	41.58	21.63	44.61	81.49	52.41	27.72	140.28	141.33	53.75	69.70	134.64	146.16	28.53	19.42	16.12	22.92	19.75
bifu4_isomer1_c4	4.04	0.43	0.04	9.58	65.19	127.43	149.99	41.63	21.61	44.24	81.55	51.49	27.57	140.26	141.03	53.70	69.55	134.61	146.19	28.53	19.41	16.15	22.82	19.73
bifu4_isomer1_c5	1.05	0.67	5.93	6.41	64.31	129.34	145.89	39.73	21.67	41.20	79.61	48.30	27.36	134.24	147.01	50.31	73.48	134.39	147.30	28.54	19.13	17.76	28.38	19.66
bifu4_isomer1_c6	1.19	0.72	4.70	5.94	65.25	133.92	140.73	39.27	22.32	38.10	82.37	49.07	27.34	135.65	143.13	53.28	73.14	134.17	145.14	28.40	19.44	17.42	29.99	19.80
bifu4_isomer1_c7	0.82	0.77	8.75	5.41	64.06	129.95	144.84	39.58	21.99	38.53	82.42	49.30	27.20	135.65	143.23	52.30	73.27	134.43	144.23	28.36	19.38	17.68	30.03	19.55
bifu4_isomer1_c8	2.19	0.83	0.87	4.87	64.25	129.07	147.23	42.22	22.01	44.38	80.39	53.72	29.54	137.52	144.48	53.10	72.20	132.95	147.47	28.49	19.42	16.63	25.67	19.87
bifu4_isomer1_c9	1.14	0.84	5.08	4.80	64.50	136.85	147.61	38.33	30.69	39.34	81.26	45.98	27.31	134.42	144.41	53.51	72.87	134.56	144.19	28.37	19.43	17.66	29.96	22.89
bifu4_isomer1_c10	0.87	0.88	7.96	4.47	64.13	129.76	144.29	39.19	21.62	40.47	82.19	46.51	27.17	135.03	144.00	52.33	73.18	134.42	144.31	28.38	19.40	17.82	30.36	19.64
bifu4_isomer1_c11	1.54	1.06	2.57	3.30	64.60	136.75	147.62	38.75	30.71	37.61	81.60	48.84	27.27	134.61	143.81	53.46	72.92	134.74	143.71	28.35	19.40	17.73	29.75	22.73
bifu4_isomer1_c12	1.42	1.25	3.16	2.40	64.54	136.39	147.12	38.25	29.14	43.77	81.74	46.24	27.14	141.86	138.93	53.68	71.39	134.20	144.96	28.38	19.49	16.61	25.86	22.82
bifu4_isomer1_c13	1.77	1.32	1.76	2.15	65.64	132.84	141.99	39.53	21.83	40.31	79.58	47.79	27.50	133.77	147.87	51.60	73.37	134.46	146.92	28.50	19.11	17.53	28.60	19.98
bifu4_isomer1_c14	1.59	1.48	2.37	1.64	63.97	134.67	148.15	42.40	25.15	37.87	82.18	49.04	28.17	133.88	141.51	46.66	73.73	134.71	142.62	28.24	19.26	21.92	29.89	16.37
bifu4_isomer1_c15	1.79	1.50	1.69	1.57	65.08	134.62	150.31	39.15	30.30	40.16	78.63	48.16	27.59	133.96	146.81	51.07	73.03	134.43	147.00	28.52	19.09	17.72	28.33	22.68
bifu4_isomer1_c16	1.33	1.70	3.67	1.12	63.83	133.56	150.53	43.30	22.01	50.89	80.17	45.89	29.24	139.74	138.55	53.23	71.87	134.88	145.38	28.51	19.35	16.96	23.55	20.90
bifu4_isomer1_c17	3.79	2.87	0.06	0.16	62.95	129.41	150.35	39.47	21.18	41.35	81.97	48.93	27.04	136.40	144.14	52.88	73.53	133.74	145.20	28.38	19.45	16.86	26.93	20.60
bifu4_isomer1_c18	3.60	3.73	0.08	0.04	63.62	139.25	148.16	47.45	23.91	47.94	80.45	48.41	28.73	140.09	139.57	51.99	74.10	131.03	151.08	29.13	21.72	17.31	23.83	16.14
bifu4_isomer1_c19	3.96	4.56	0.04	0.01	63.63	127.88	149.07	38.51	24.39	34.67	83.20	50.80	28.36	133.89	141.29	47.18	73.92	134.70	142.69	28.24	19.30	21.85	29.34	19.42
bifu4_isomer1_c20	3.96	4.73	0.04	0.01	63.95	136.52	146.37	42.70	23.70	43.08	82.53	45.79	27.12	141.67	139.33	51.90	73.25	130.85	151.62	29.07	21.87	16.43	25.33	16.29
bifu4_isomer1_c21	5.43	5.28	0.00	0.00	64.14	129.72	145.70	39.55	21.70	40.88	80.07	48.18	27.12	132.82	149.47	49.48	77.15	133.52	152.15	29.51	21.78	18.62	28.27	19.70
bifu4_isomer1_c22	4.97	5.35	0.01	0.00	64.27	136.60	147.86	37.99	30.73	39.89	81.48	45.89	27.15	133.84	145.49	51.51	74.27	131.54	150.66	29.04	22.20	18.04	29.78	22.84
bifu4_isomer1_c23	5.29	6.23	0.00	0.00	64.42	136.71	147.73	38.43	30.94	37.95	81.87	48.70	27.12	134.23	144.69	51.46	74.24	131.58	150.77	29.03	22.32	18.07	29.62	22.64
Boltzmann average					**64.18**	**132.75**	**147.89**	**42.07**	**23.54**	**43.17**	**81.17**	**48.86**	**27.71**	**138.79**	**141.44**	**52.99**	**72.03**	**134.44**	**145.33**	**28.44**	**19.38**	**17.03**	**26.17**	**18.92**

	ΔE	ΔG	pop-ΔE	pop-ΔG	Calculated ^{13}C-chemical shifts for (7R,13S)-isomer (b3lyp/6-311++G(2d,p)/IEFPCM/$CHCl_3$)																			
	**ΔE**	**ΔG**	**pop-ΔE**	**pop-ΔG**	1	2	3	4	5	6	7	8	9	10	11	12	13	14	15	16	17	18	19	20
bifu4_isomer2_c1	0.00	0.16	35.05	31.81	64.82	131.26	154.07	47.09	28.76	43.30	79.44	52.97	29.73	138.23	142.47	54.08	69.66	134.79	145.98	28.52	19.40	16.47	26.25	16.36
bifu4_isomer2_c2	0.00	0.00	34.86	41.86	64.97	131.42	153.03	48.62	28.54	43.38	79.36	52.99	29.78	138.16	142.47	54.02	69.72	134.79	145.94	28.52	19.39	16.52	26.30	17.05
bifu4_isomer2_c3	0.17	0.30	26.50	25.38	64.93	131.25	153.06	48.52	28.95	45.00	79.19	50.15	26.07	138.19	142.61	53.67	69.73	134.83	145.80	28.51	19.42	16.43	27.47	17.02
bifu4_isomer2_c4	2.01	4.95	1.19	0.01	64.57	135.86	146.33	45.63	24.01	42.31	80.26	48.47	26.28	133.42	142.03	48.97	70.80	132.28	148.00	28.49	19.48	20.34	27.20	15.21
bifu4_isomer2_c5	2.15	2.42	0.93	0.70	64.87	126.26	153.60	41.74	25.97	39.73	80.71	52.79	26.80	139.29	142.09	53.69	69.52	134.80	146.01	28.53	19.40	16.55	29.80	19.42
bifu4_isomer2_c6	2.78	3.92	0.32	0.06	64.69	132.63	151.65	46.23	23.62	45.11	79.85	47.10	28.10	137.62	146.41	50.56	75.69	133.41	146.35	28.63	19.03	20.60	24.20	16.10
bifu4_isomer2_c7	2.90	4.05	0.26	0.04	65.00	131.46	153.23	47.13	29.87	50.08	82.56	41.77	27.58	136.31	142.56	53.73	72.23	136.06	143.70	28.46	19.50	16.98	30.61	16.45
bifu4_isomer2_c8	2.91	4.70	0.26	0.02	64.84	131.60	152.68	48.42	31.28	46.65	82.26	42.73	27.77	137.05	141.29	50.55	75.43	136.38	140.75	28.32	19.04	20.05	31.89	16.92
bifu4_isomer2_c9	3.04	3.71	0.21	0.08	64.75	133.26	150.15	47.21	27.30	44.32	79.49	42.85	26.82	140.06	140.48	50.87	72.54	136.62	141.55	28.27	19.07	20.62	31.74	16.39
bifu4_isomer2_c10	3.10	4.29	0.19	0.03	64.71	129.90	152.47	48.53	28.22	52.63	81.52	40.66	28.89	139.19	138.75	50.61	74.99	136.71	140.63	28.32	18.96	20.85	29.80	17.47
bifu4_isomer2_c11	3.23	6.04	0.15	0.00	64.07	127.28	157.43	46.98	28.10	44.22	80.12	46.09	29.51	130.64	151.11	50.89	77.78	139.38	141.08	28.29	19.85	19.89	31.42	16.51
bifu4_isomer2_c12	4.18	5.19	0.03	0.01	64.70	132.64	151.61	46.30	23.62	45.15	79.86	47.13	28.17	136.40	145.61	53.06	74.24	135.02	150.79	29.32	20.95	16.39	24.30	16.10
bifu4_isomer2_c13	4.25	5.42	0.03	0.00	64.91	131.29	153.07	48.63	30.73	49.58	79.38	44.07	28.05	133.92	147.67	51.51	75.07	134.29	150.37	29.35	20.85	16.81	26.32	17.02
bifu4_isomer2_c14	4.70	6.67	0.01	0.00	65.21	132.60	154.86	42.73	34.07	47.37	79.62	42.97	29.37	139.30	141.28	49.01	75.02	134.46	145.44	28.54	19.08	20.73	25.21	21.30
bifu4_isomer2_c15	5.20	9.81	0.01	0.00	63.68	128.40	149.10	38.40	24.52	34.85	83.38	51.65	27.40	136.91	141.24	49.29	74.96	133.76	143.17	28.47	19.12	20.38	29.53	19.53
bifu4_isomer2_c16	5.74	8.76	0.00	0.00	63.96	127.42	157.57	47.08	28.01	47.88	80.46	41.32	28.74	130.87	151.16	50.75	81.04	136.80	147.44	29.40	20.59	19.57	31.70	16.47
bifu4_isomer2_c17	5.83	8.99	0.00	0.00	63.91	127.80	157.20	47.01	27.90	46.43	80.11	45.07	29.06	130.82	151.32	50.60	81.18	136.70	147.59	29.39	20.60	19.63	28.24	16.41
bifu4_isomer2_c18	7.04	10.69	0.00	0.00	64.65	132.58	149.99	46.38	29.11	47.65	80.28	41.18	29.16	135.29	146.03	50.31	81.79	130.80	151.06	29.08	21.45	20.77	31.45	16.66
bifu4_isomer2_c19	7.18	11.02	0.00	0.00	66.54	134.93	148.74	41.01	27.23	36.95	81.51	50.53	26.97	125.71	150.24	48.12	72.94	135.94	148.43	29.36	20.82	19.60	28.79	17.14
bifu4_isomer2_c20	7.23	10.94	0.00	0.00	64.94	130.12	151.79	47.01	27.03	47.02	79.88	39.26	28.51	134.16	147.70	49.77	80.90	136.97	146.73	29.61	20.22	20.86	33.58	17.11
bifu4_isomer2_c21	7.35	11.15	0.00	0.00	64.67	133.00	149.42	46.34	28.91	46.27	79.93	44.84	29.50	135.12	146.29	50.27	81.84	130.75	151.12	29.07	21.45	20.78	28.00	16.62
Boltzmann average					**64.91**	**131.29**	**153.37**	**48.06**	**28.69**	**43.75**	**79.36**	**52.24**	**28.80**	**138.20**	**142.50**	**53.94**	**69.71**	**134.80**	**145.91**	**28.52**	**19.40**	**16.49**	**26.61**	**16.84**
